# In *Arabidopsis thaliana* codon volatility scores reflect GC3 composition rather than selective pressure

**DOI:** 10.1186/1756-0500-5-359

**Published:** 2012-07-17

**Authors:** Mary J O'Connell, Aisling M Doyle, Thomas E Juenger, Mark TA Donoghue, Channa Keshavaiah, Reetu Tuteja, Charles Spillane

**Affiliations:** 1 Bioinformatics and Molecular Evolution Group, School of Biotechnology, Dublin City University, Dublin 9, Ireland; 2 Genetics and Biotechnology Lab, Department of Biochemistry, Lee Maltings 2.10, University College Cork (UCC), Cork, Ireland; 3 Section of Integrative Biology and Institute for Cellular and Molecular Biology, University of Texas at Austin, Austin, TX, USA; 4 Genetics and Biotechnology Lab, Plant and AgriBiosciences Research Centre, Aras de Brun C306, National University of Ireland Galway (NUIG), Galway, Ireland

## Abstract

**Background:**

Synonymous codon usage bias has typically been correlated with, and attributed to translational efficiency. However, there are other pressures on genomic sequence composition that can affect codon usage patterns such as mutational biases. This study provides an analysis of the codon usage patterns in *Arabidopsis thaliana* in relation to gene expression levels, codon volatility, mutational biases and selective pressures.

**Results:**

We have performed synonymous codon usage and codon volatility analyses for all genes in the *A. thaliana* genome. In contrast to reports for species from other kingdoms, we find that neither codon usage nor volatility are correlated with selection pressure (as measured by *dN/dS*), nor with gene expression levels on a genome wide level. Our results show that codon volatility and usage are not synonymous, rather that they are correlated with the abundance of G and C at the third codon position (GC3).

**Conclusions:**

Our results indicate that while the *A. thaliana* genome shows evidence for synonymous codon usage bias, this is not related to the expression levels of its constituent genes. Neither codon volatility nor codon usage are correlated with expression levels or selective pressures but, because they are directly related to the composition of G and C at the third codon position, they are the result of mutational bias. Therefore, in *A. thaliana* codon volatility and usage do not result from selection for translation efficiency or protein functional shift as measured by positive selection.

## Background

Codon-based metrics are widely used to analyse selective pressures operating on genes and genomes [1,2]. For instance, the relative rate ratio test of non-synonymous to synonymous substitutions (dN/dS) can provide a measure of the type of selection operating on a particular gene [3]. Such dN/dS metrics can be used to identify selective pressure variation (on gene families) within sequenced genes or genomes.

In the model plant *Arabidopsis thaliana*, dN/dS based approaches have been used to identify genes that are rapidly evolving and have undergone positive selective pressure, such as the mate selectivity genes [4], the pollen-specific oleosin-like proteins (oleopollenins) [5], the methylthioalkylmalate synthases (*MAM* genes) [6], the *MEDEA* gene [7]. dN/dS based approaches have also been used to study the diversification of genes encoding different cytochrome P450 enzymes [8] and the evolution of auxin signalling pathways [9]. The link between positive selection and protein functional shift has been confirmed using empirical data [10,11]. The identification of signatures of positive selection in protein coding genes has become increasingly relevant for understanding protein specificity and function [10,11]. All dN/dS-based comparative approaches require extensive sequence data for the genes under scrutiny, ideally from a range of individuals across multiple species.

To overcome the requirements for extensive sequence data for analysis of selection operating on genes, Plotkin et al. (2004) proposed a “codon volatility” approach for detecting selection using a single sequenced genome [12]. Codon volatility measures the different number of mutations required for synonymous codons to be transformed into codons encoding a different amino acid and was proposed to reflect the selective pressure acting on genes [12-14]. Calculation of codon volatility is rapid, depending only on DNA sequence data and requiring a single genome rather than multiple. The “volatility” of a codon is defined as the probability that a random point mutation in a codon can generate a non-synonymous amino acid change [12-14]. For example, for the codon ‘AGA’ (coding for arginine) if one allows a point mutation in any of the three positions of the codon, eight different ancestral codons may have existed to give rise to ‘AGA’ (stop codons as potential ancestor codons are disallowed in the codon volatility calculation). The next step to determine a codon volatility metric is to determine whether the point mutation causes a synonymous change or non-synonymous change. In the example, ‘AGA’, two of the eight ancestral codons may have encoded arginine, while the other six codons coded for a different amino acid. For this codon the codon volatility would be 6/8. The sum of all the volatilities in a coding sequence determines the overall codon volatility of the gene. An overall codon volatility P-value is then assigned to each gene in the genome to provide a significance-based metric indicating whether a given gene is more (P close to 1) or less (P close to 0) volatile than all of the other genes in the genome. It was initially proposed that a higher-than-expected mean codon volatility for a gene could indicate that positive selection for non-synonymous changes has acted on this gene in the recent past [12-14]. However, subsequent analyses of this metric determined that it did not reflect the selective pressure at work on a gene. Rather, it has since been suggested that codon volatility values are mostly measuring differences in four amino acid families (glycine, leucine, arginine, and serine) or variation in codon usage bias [15-18]. Codon volatility has been strongly criticised as a method for detecting positive selection and we are in agreement with the critiques of codon volatility that have been indicated to date [15-22].

Many of the critiques of the codon volatility method suggest that it is essentially a measure of codon usage bias [23]. For example, an analysis of the yeast genome demonstrated that correlations between codon volatility and dN/dS (or dN) are likely to be due to a correlation between dN/dS (or dN) and translational codon bias [24]. Plotkin et al. (2006) responded to such criticisms by reiterating that codon volatility P-values are intrinsically relative, and that the codon volatility method can only conclude that some genes are under more positive, or less negative, selection than others. To the authors’ knowledge, codon volatility has not been tested in any plant genome, nor has its relationship with codon usage bias been investigated in any plant species. In most prokaryotes, and many eukaryotes, variation in synonymous codon usage may reflect the effects of selection for translational efficiency [25,26], as shown in bacteria [27], *H. pylori*[28], yeast [29], *C. elegans*[30], Drosophila [31] and mammals [32]. It has also been suggested to occur due to selection for translational efficiency in plant organelles [33] and nuclear genomes, including those of *Arabidopsis thaliana* and its sister species *Arabidopsis lyrata*[34-38].

There are also cases where codon usage patterns do not seem to be simply due to tRNA abundance and selective pressure related to increase translational efficiency. For instance, synonymous codon usage in mammals is correlated with GC content of the region in which the gene is located, and is generally thought to reflect mutational bias [39]. However, it has been observed that high GC content increases mammalian mRNA levels [40] and indeed previous studies in species such as *Arabidopsis thaliana* have also suggested a correlation between codon usage bias and gene expression levels [41]. Between different plant species, increased GC content in coding sequences is observed in monocots [42-44], and plant genes can display a context dependency regarding codon usage patterns [38].

In this study we have tested for the role of mutational bias and selective pressures (translational efficiency and positive selection), in defining the observed codon usage patterns and codon volatility levels observed in the genome of the model plant *Arabidopsis thaliana*. Our approach is based on a comparative analysis of codon usage bias and codon volatility distributions using homologs in the genomes of the sister species, *Brassica oleracea* and *Arabidopsis lyrata*.

## Methods

### Codon usage analysis

The complete genome of *A. thaliana* was downloaded from the TAIR website [45]. We did not attempt to remove any sequences from the data set based on their sequence length, assignment of function or status as hypothetical or other. The dataset consisted of 28,952 genes in total.

Two methods of codon usage analysis were applied to the data. The package CODONW [46] was used to determine the percentage inertia of the various axes. The software package GCUA [47] was then used to determine all relative synonymous codon usage (RSCU) values, the effective number of codons and the G + C base composition at the third position of synonymously degenerate codons (GC3 composition). GCUA program was modified for the whole genome sequence. Codons that have a synonymous alternative (59 codons) were used in the analysis. Correspondence analysis of the RSCU values was performed to identify the axes that contributed most significantly to the variation observed in RSCU values. Every gene in the *A. thaliana* genome was summarized in 59-dimensional space and the axes contributing most significantly to the distribution of RSCU values were identified using multivariate analysis. These axes were named Axis 1 and Axis 2, as they contributed 8% and 5% respectively of the inertia in the dataset whereas no other axes made significant contributions to the inertia.

### Codon Volatility analysis of the *Arabidopsis* genome

The *A. thaliana* coding sequences were downloaded from TAIR (ATH1_cds, February 2004). A total of 29,157 predicted protein-coding genes were available for *A. thaliana*. We analysed the *Arabidopsis thaliana* genome using a transition:transversion ratio of 4.1 (Kappa). The volatilities of individual codons were calculated and added across the coding sequence as suggested by Plotkin *et al.* using the source code from the online Codon Volatility Computation Server [48]. Those *A. thaliana* genes with elevated volatilities (*P-*value <10^-6^) were selected for further analysis.

### Comparative dN/dS study

Preliminary *Brassica oleracea* sequence data was obtained from the former The Institute for Genomic Research (TIGR) now The J. Craig Venter Institute (JCVI) [49]. A total of 454,274 shotgun sequencing reads were available at the time of the analysis (June 2003). The reads were clustered and assembled into contigs using the TIGR Gene Indices clustering tools (TGICL) [50] using default parameters throughout. The resulting dataset (367,108 sequences containing 325,017 singleton sequences and 42,091 clusters) had less redundancy than the *B. oleracea* preliminary shotgun sequences and some longer consensus contig sequences were created. The *B. oleracea* sequences were aligned against the *A. thaliana* coding sequences (CDS) using the BLASTN program (expected value < 10^-15^). 53,051 *B. oleracea* sequences had significant matches to 18,828 distinct *A. thaliana* CDS. The protein product of the best *Arabidopsis* hit was used as a model to extract the *B. oleracea* coding sequence using the Genewise program [51]. In most cases, the *B. oleracea* sequences consisted of partial gene sequences. For each *ArabidopsisBrassica* ortholog gene pair, the two translation products were then aligned using the Smith-Waterman algorithm [52], and the resulting alignment was used as a guide to align the nucleotide sequences. The codeml program (from the PAML package v3.13) was used to calculate the dN/dS ratio between each pair of sequence using the one-ratio model M0. The dN/dS score for five of the *A. thaliana* elevated volatility genes was determined (remainder of the genes did not have a *Brassica* ortholog). Two sources of *A. lyrata* sequence were used in this study. Prior to the publication of the draft *A. lyrata* genome, four elevated volatility genes were chosen at random from a total of eleven and were amplified, cloned and sequenced. We subsequently obtained the draft *A. lyrata* genomic sequences courtesy of the Joint Genome Institute (DOE JGI) [53] and identified nine out of these eleven by the reciprocal best BLASTN hit approach. The four cloned genes, together with the nine sequences obtained from the draft genome, accounted for ten out of the eleven elevated volatility genes we had identified whilst we were unsuccessful in cloning one gene (At4g15430) or identifying it on early genomic scaffold sequence data for *A. lyrata.* The protein product of the best *A. thaliana* hit was used as a model to predict the *A. lyrata* coding sequence [51]. The online EMBOSS Transeq program was used to translate the *A. lyrata* cDNA in all six reading frames [54]. The chosen protein sequence was then aligned to the *A. thaliana* ortholog protein using MUSCLE from EBI [55]. TranAlign was used to align the *A. thaliana* and *A. lyrata* CDS according to the aligned gapped protein sequences [56]. Pairwise analysis as described above was used to calculate the dN/dS ratio of the four cloned and nine obtained *A. lyrata* genes [57].

### Plant materials

*A. thaliana* (Columbia / Col-0 accession) and a relative species *A. lyrata* (255_S7, 255_S8, 255_S9 seeds provided by Dr. Karl Schmid, University of Hohenheim, Germany) were obtained. Seeds from each accession were sterilized in 500 μl of seed sterilization solution (16.6% (v/v) sodium hypochlorite (Water Technology Limited, Ireland), 10% (v/v) triton® X-100 (Sigma, Germany) and made up to an appropriate volume with sterilized Millipore water. After inverting the tube for 15 minutes the sterilization solution was removed and the seeds were washed three times in 500 μl Millipore water for 15 minutes. The seeds were left in the final water rinse and placed in a fridge at + 4°C for four days. Seeds were allowed to germinate on Murashige and Skoog Basal Medium (Sigma, Germany) (adjusted pH to 5.8 using KOH) in a Percival tissue culture growth cabinet (Percival Scientific Inc., Germany) with the following photoperiod: 16-hr light (21°C)/8-hr dark cycles (18°C). After two weeks, the seedlings were transferred to Westland multipurpose potting compost mix (Westland multipurpose potting compost (Westland, UK) 70% (v/v) with added vermiculite medium (Sinclair, UK) 15% (v/v) and perlite standard (Sinclair, UK) 15% (v/v). The seedlings were transferred to individual pots and covered for 10 days. Plants were grown in purpose built *Arabidopsis* growth chambers (Cambridge Scientific, UK) under the following growth conditions with 16-hr light (21°C)/8-hr dark cycles (18°C).

### Gene expression analysis

Three replicate plants of *A. thaliana* accession Columbia (Col-0) were grown using standard growth chamber conditions (150 μmol m-2 s-1 PPFD, 14 h light/10 h dark, 20°C, with a constant RH of 50%). After 20 days, several fully expanded rosette leaves were harvested from each replicate plant and placed in RNAlater (Ambion, Austin, TX, USA) and total RNA was extracted using the Qiagen RNeasy kit (Germantown, MD, USA). The integrity of total RNA was qualified by Agilent Bioanalyzer 2100 capillary electrophoresis and used for preparation of biotin-labeled targets (cRNA) using a MessageAmp™ II-based protocol (Ambion Inc., Austin, TX). Labelled cRNA was fragmented and used for array hybridization and washing according to the standard Affymetrix protocol. Raw intensity measures from .CEL files were imported into the R Statistical environment using the Affy procedure and gene expression measures generated using the RMA function (background corrected, log2 transformed, quantile normalized, median-polished summary). The average of the three Col-0 replicate RMA expression measures was used for all subsequent analyses.

### DNA/RNA isolation, cDNA synthesis, cloning, PCR and sequencing

Genomic DNA was extracted from *A. thaliana* and wild relative *A. lyrata*. 100 mg of plant tissue (rosette leaf, stem, flower, bud, cauline leaf) was harvested and snap frozen in liquid nitrogen. The tissue was ground twice in a TissueLyser (Qiagen, Germany) for 1 minute at 30 Hz and snap frozen for 2 minutes between each lysis. DNA was extracted from this using the DNeasy Plant Mini Kit (Qiagen, Germany) following the manufacturer’s instructions and autoclaved Millipore water was used to elute the DNA twice in 50 μl. 1 μl of the isolated DNA was used in the downstream PCR reactions.

Total RNA was isolated from at least three weeks post germination *A. lyrata* tissue (rosette leaf, shoot, flower, bud, cauline leaf) following the protocol form the RNeasy Plant Mini Kit (Qiagen, Germany). The RNA was eluted twice in 40 μl RNase-free water. Reverse transcription was used to synthesise cDNA from the *A. lyrata* mixed organ RNA using the Quantitect Reverse Transcription Kit (Qiagen, Germany). The Quantitect Reverse Transcription Kit protocol was followed. The cDNA samples were diluted 1:10 in sterile Millipore water and 3 μl was used per PCR reaction.

PCRs were carried out using Expand™ High Fidelity PCR system (Roche, Germany). The reactions were carried out in 25 μl volumes in a Dyad Disciple™ Peltier Thermal Cycler (MJ Research), containing 3 μl of template cDNA or 1 μl (~5 ng) of template DNA. PCR reaction mixtures as recommended by the Expand High Fidelity PCR system (Roche) were used. The samples were denatured at 95°C for 2 minutes and then subject to 30 cycles of denaturation at 95°C for 30 seconds, annealing temperature of 55°C for 45 seconds and polymerization at 72°C for 1 minute per kb plus a final extension of 72°C for 7 minutes. 8 μl aliquot of amplified product was analysed on a 1% w/v agarose gel.

Single bands were purified using the QIAquick PCR purification kit (QIAGEN, Germany) following handbook guidelines and eluted in 30 μl of autoclaved Millipore water. Multiple bands were excised and purified using the QIAquick Gel extraction Kit (QIAGEN, Germany) following the kit handbook guidelines. The purified *A. lyrata* products were cloned into the pCR-Blunt II-TOPO vector using the Zero Blunt TOPO PCR cloning kit (Invitrogen, Carlsbad, CA) and transformed into *E. coli* TOP10 chemically competent cells (Invitrogen, Carlsbad, CA). Colony PCR was used to analyse transformants for correct sized insert. The universal M13F (−20) and M13R vector derived primer pair were used. A sterile tip was used to isolate the corner of one single colony and the PCR reaction were performed in 25 μl volume using the GoTaq® Flexi DNA polymerase (Promega, WI) enzyme following manufactures guidelines. A single bacterial colony containing a plasmid was used to inoculate 5 ml of LB media (10 g/l tryptone peptone, 5 g/l yeast extract, 10 g/l NaCl, adjust pH to 7.0 using 1 M NaOH) containing Kanamycin (50 g/ml) antibiotic. The culture was grown for 16 hr at 37°C with shaking (225 rpm) in an orbital shaker, following which the plasmid DNA was extracted from the bacterial cells using the QIAprep Spin Miniprep Kit (Qiagen, Germany) as per the manufacturer's instructions. Plasmid miniprep DNA was isolated using the QIAprep Mini-prep Kit (Qiagen, Germany). 100 ng/μl of the plasmid was sent to Macrogen (Korea) for single extension sequencing [58]. For each gene, up to four independent plasmid clones were sequenced in both directions, using M13F (−20) and M13R universal primers as sequencing primers. DNASTAR software package (DNASTAR, Madison, WI) was used for sequence assembly and chromatographs were manually checked to confirm mis-called nucleotides between reads.

### Primers for cloning and sequencing of elevated volatility candidate genes in *A. lyrata* and *Arabidopsis* accessions

Four genes with elevated codon volatility were chosen at random for cloning and sequencing; At1g62240, At3g21420, At3g28780 and At5g59990. The primers for the genes are as follows: At1g62240_F 5'-GCC AAT GGC CTA ATG ATG CTG-3', At1g62240_R 5'-GAG TCC TCA ATG GCC ACG GG-3'; At1g64370_F 5'-CCA CTA GCA AAT GTA GCC TAG C-3', At1g64370_R 5'-CCA CCG TAT ATA CGA TGG TAG-3'; At3g28780_CV_F2 5'-TCT TTT ACA TAT CTG ACA AAA CAA TGG-3', At3g28780_CV_R2 5'-GGT TTT TGA CCT CTG GGT CT-3' and At5g59990_CV_F2 5'-GTG TGG ACA CGT CAG CAC TT-3', At5g59990_CV_R2 5'-TTG AGG AAG TGA TTA GCA GAG AAA-3'.

## Results

### Codon volatility analyses of *A. thaliana* genes

To determine the codon volatility scores for all genes in the *A. thaliana* genome, all of the protein coding sequences in the *Arabidopsis genome* (29,157 protein coding genes from The *Arabidopsis* Information Resource (TAIR)) were used to calculate *P*-values representing the degree of codon volatility for each gene. Genes with *P*-values < 10^-6^ were considered to be most highly volatile [9]. Using this criterion and a Kappa value = 4.1 (representing a transition/transversion ratio for the *A. thaliana* genome), 2,181 of the 29,157 genes were identified as having significantly elevated codon volatilities. An additional file provides information on the genes with significantly elevated codon volatilities [see Additional file 1.

### Genome wide codon volatility versus dN/dS analysis: *Arabidopsis thaliana* vs *Brassica oleracea* comparison

**Figure 1:**
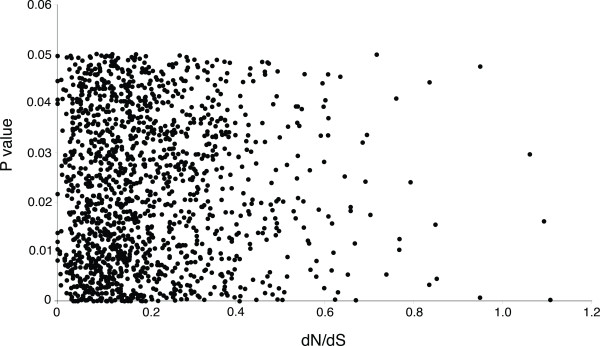
**Relationship between selection pressure (as measured by dN/dS) and codon volatility.** The 2,181 significant codon volatility candidates are compared to their dN/dS value. Pairwise dN/dS calculations between *A. thaliana* and *B. oleracea* on the x-axis versus the 2,181 significant codon volatility candidates (P < 0.050) on the y-axis.

**Figure 2:**
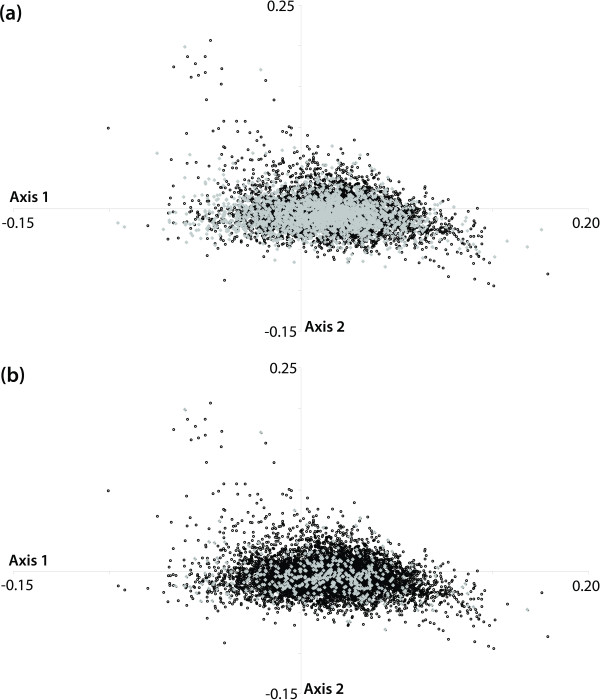
**Synonymous codon usage distribution in *A. thaliana*in comparison with codon volatility and highly expressed gene data.** The two major axes are shown here, Axis 1 and Axis 2. The darker points on the plots represent the codon usage values for each of the 18,828 genes. (**a**) The lighter points overlayed on the codon usage distribution are those genes with significant codon volatility *P*- values (2,181). (**b**) The lighter points overlayed are those genes that are highly expressed.

The codon volatility method was originally proposed as a method for identifying selective pressure variation [12]. If the codon volatility method can identify genes under positive selection in the *Arabidopsis thaliana* genome, codon volatility scores for each gene should be positively correlated with dN/dS values greater than 1 across all genes in the genome. To determine if this is the case, we conducted a genome wide dN/dS analysis of the *A. thaliana* genome using comparisons with *Brassica oleracea* shotgun sequence data. Such an analysis required the identification of likely orthologous pairs between the *A. thaliana* and available *B. oleracea* sequences. Orthologous pairs were identified using reciprocal BLASTN resulting in 53,051 *B. oleracea* sequences that matched significantly with 18,828 distinct *A. thaliana* coding DNA sequences (CDSs). The *B. oleracea* shotgun sequence consisted of genomic sequence reads of ~650 nucleotides (nt) in length [59]. Since the *Brassica* shotgun sequences were short (~650 nt), most of the larger *A. thaliana* CDSs had partial *Brassica* orthologous regions. The average coverage was 30%. To determine the dN/dS values of all 18,828 pairwise alignments of *A. thaliana:B. oleracea* orthologs (representing 18,828 independent gene models), the dN/dS ratio was calculated for each of the likely orthologous pair using the one-ratio model M0 from the codeml program in the PAML package v3.13 [2]. These dN/dS values were then plotted against the 2,181 (P < 0.050) significant codon volatility *P*-values to determine if there is any correlation between codon volatility values and dN/dS values (Figure 1). A correlation between lower *P*-values and higher dN/dS values would indicate that enhanced codon volatility corresponds to selective pressures of dN/dS > 1, but no such correlation was found (Figure 1). In the region of Figure 2 where dN/dS is greater than 1, we would expect to find an upper left-lower right correlation between the dN/dS values in this region and the volatility scores, thereby showing that at higher dN/dS values the significance of volatility score increases to 0. However, no such correlation is seen. Our results indicate that codon volatility scores are not positively correlated with dN/dS values across the *A. thaliana* genome, and hence that codon volatility scores are unlikely surrogate metrics for rapidly evolving genes in this genome.

### Genome wide codon volatility versus dN/dS analysis: a comparison of *A. thaliana* vs *A. lyrata*

To further investigate any possible correlation between dN/dS values and codon volatility we selected the most highly volatile genes for dN/dS comparisons between orthologs in *Arabidopsis thaliana* vs *Arabidopsis lyrata*. Eleven genes were selected as the top cohort of genes from the overall highly volatile group of 2,181 genes (most highly volatile based on *P*-value of 10^-6^ and kappa value of 4.1) (Table 1).

**Table 1:** **Highest Volatility Candidates in *Arabidopsis thaliana*and dN/dS comparative sequence analysis to*B. oleracea*and*A. lyrata***

			**Volatility Analysis**	**dN/dS Selection Analysis**			
			***A. thaliana***	***A. thaliana vs B. oleraca***		***A. thaliana vs A. lyrata***	
**Gene**	**Description**	**CDS (bp)**	***P*-values**	**dN/dS**	**(%CDS)**	**dN/dS**	**(%CDS)**
At1g62240	expressed protein	684	0.000000006			0.7975*	(100)
At1g64370	expressed protein	537	0.000000323			0.2567	(94)
At1g69440	PAZ domain-containing protein	2973	0.000008714	0.1098	(15)	0.0910	(93)
At2g27380	proline-rich family protein	2286	0.000000000			0.0412	(25)
At3g21420	oxidoreductase	1095			(22)	0.1173*	(88)
						0.0913	(84)
At3g28780	glycine-rich protein	1845				0.4217*	(95)
						0.2545	(53)
At4g15430	early-responsive to dehydration	2271	0.000004120	0.3808	(17)		
At4g31590	glycosyl transferase family 2	2079	0.000004420	0.2099	(29)	0.1028	(100)
At4g32420	peptidyl-prolyl cis-trans isomerase	2514	0.000005314			0.2893	(98)
At5g07570	glycine/proline-rich protein	4515	0.000001603			0.4833	(71)
At5g59990	expressed protein	726			(55)	0.1869*	(90)
						0.2227	(100)

The 11 most volatile genes in the *A. thaliana* genome with their corresponding volatility *P*- values (P < 10^-6^). Conventional analyses using pairwise dN/dS ratios (CODEML M0 from PAML). Displayed are those comparisons that were possible between *A. thaliana* and homologous sequences, using shotgun sequencing reads (*B. oleracea*), close to full length sequences (*A. lyrata)* and four cloned genes (*A. lyrata)* marked by *.

To determine if each of the eleven most highly codon volatile *A. thaliana* genes were rapidly evolving, comparative sequence data from the sister species *Arabidopsis lyrata* was used to calculate dN/dS ratios for each gene. The *A. lyrata* sequences were obtained from a combination of PCR amplification/sequencing from cDNA (four of the smaller coding regions indicated by * in Table 1), and from genome sequence data. None of the eleven most highly volatile genes tested in comparison with *A. lyrata* had dN/dS > 1 (observed values ranged from 0.0412 to 0.7975) therefore there is no evidence that they are evolving rapidly (Table 1). Partial genome sequences were available from *B. oleracea* for five of these genes, and dN/dS analysis using these sequences did not show any evidence for dN/dS > 1 as would be associated with rapidly evolving genes either (Table 1). These results demonstrate that codon volatility in *A. thaliana* is not synonymous with rapid evolution.

### Codon volatility divergence between gene paralogs in *Arabidopsis thaliana*

Paralogous genes with similar sequence identity could exhibit similar levels of codon volatility if they retain similar functions, whereas paralogs with widely divergent codon volatility could be indicative of divergence in gene function. To determine whether any of the high codon volatility genes had paralogs in the *A. thaliana* genome, paralogons were identified in the “Paralogons in *Arabidopsis thaliana* Database” using AGI gene names as queries [60,61]. Only paralogs from duplicate blocks of greater than 6 (sm >6) were considered. Based on this criterion, nine of the high volatility candidates had no apparent paralogs. The remaining two genes (At4g15430, At4g31590) each had a single paralog, At3g21620 and At2g24630 respectively (Table 2). Each of these paralogs was less volatile than its corresponding elevated volatility paralog, indicating that codon volatility between these duplicates has become asymmetric following gene duplication. While this indicates that pairs of paralogous genes can differ in terms of their codon volatility, the biological meaning of this divergence of codon volatility measures between paralogs remains unclear.

**Table 2:** High codon volatility candidates and their paralogs

	**Gene**	**Function**	**Volatility (*P-*value)**
*Candidate*	At4g15430	early-responsive to dehydration protein-related	0.0000041
Paralog	At3g21620	early-responsive to dehydration protein-related	0.0720094
*Candidate*	At4g31590	glycosyl transferase family 2 protein	0.0000044
Paralog	At2g24630	glycosyl transferase family 2 protein	0.8029084

Comparison of volatility *P*-values for most volatile *Arabidopsis* genes and their paralogs. Paralog information was extracted from the ‘Paralogons in Arabidopsis thaliana’ database (http://wolfe.gen.tcd.ie/athal/dup). The first two columns display the candidate gene, its paralog, and a brief description of their function. The last column is the codon volatility P-value.

This analysis of codon volatility and selective pressure between pairs of likely orthologs between *A. thaliana* and *B. oleracea*, and *A. thaliana* and *A. lyrata*, indicates that in these plant species, elevated codon volatility is not an indication of rapid sequence evolution and hence is not dependent upon selective pressure. The *A. thaliana* genes with the highest codon volatility are not under positive selection as demonstrated by dN/dS generated by comparative sequence analysis and their volatility is therefore caused by some other factor or factors.

### Codon usage bias versus codon volatility scores

As codon volatility is not necessarily associated with rapid gene evolution, we sought to decipher what other factors could be causing the differences in volatility that we observed. In four yeast species *(Saccharomyces cerevisiae, Saccharomyces paradoxus, Saccharomyces mikatae,* and *Saccharomyces bayanus)* it has been shown that codon volatility is correlated with the extent of translational codon usage bias [24]. To determine whether such a correlation is also observed in the multicellular eukaryote *A. thaliana*, we conducted a genome-wide comparison of codon volatility scores with relative synonymous codon usage (RSCU) values.

Correspondence analysis of RSCU values was performed on all genes in the *A. thaliana* genome and the two most significant axes selected for further analysis (Figure 2) [47]. The first axis accounted for 8% of the total inertia of the 59-dimensional space. The second axis accounted for 5% of the total inertia, and no other axis accounted for any significant level, i.e. no other axis had >5% inertia [62]. Most of the variation in the second axis can be accounted for by considering the amino acid composition of the most extreme genes that in all cases are proline-rich proteins. Axis 2 is therefore accounted for by amino acid bias. Axis 1 is responsible for the majority of the remaining variation (Figure 2A).

Our results indicate that the 2,181 genes with significantly elevated codon volatilities fall completely within the normal codon usage distribution (Figure 2A), indicating that the 2,181 genes with significant codon volatility values are not those genes with the most extreme codon usage. The mean and standard deviation are identical, indicating that codon usage and the codon volatility follow exactly the same distribution. In *A. thaliana*, if codon volatility is simply another measure of biased codon usage it would be expected that the significant codon volatility values would correspond to the significant codon usage values, but this is not the case. Therefore, codon usage and codon volatility are not synonymous within the *A. thaliana* genome.

### Codon usage bias versus gene expression levels

The translational efficiency model for codon usage bias is supported by strong correlations between codon usage and gene expression levels in various taxa [35]. To determine whether codon volatility or codon usage values are correlated with expression levels in *A. thaliana*, whole genome transcript expression level data was used (from the Affymetrix ATH1 array from RNA harvested from fully-expanded rosette leaves from the Columbia accession Col-0) for comparison with codon volatility scores. Comparative analysis of 16,162 unique gene expression data points in conjunction with the codon volatility scores for these 16,162 genes indicated no correlation between gene expression level and codon volatility or codon usage (Figure 2B).

In particular, we tested if any correlation exists between the most highly expressed genes (2,703) and the most significant codon volatility and/or the most extreme codon usage bias. The coverage across the *A. thaliana* genome for this expression dataset analysis is approximately 90%. The average expression value is 6.44 and the standard deviation across the dataset is 2.02. The most highly expressed genes can be seen in Figure 2B as the paler data points. The highly expressed genes do not display characteristic codon usage patterns (Figure 2B). Our results indicate that there is no significant correlation between codon volatility or codon usage bias and gene expression levels in the *A. thaliana* genome. Therefore, we find no support for the translational efficiency model to explain either codon usage bias or codon volatility in *A. thaliana*.

### Codon volatility and codon usage bias versus GC mutational bias

**Figure 3:**
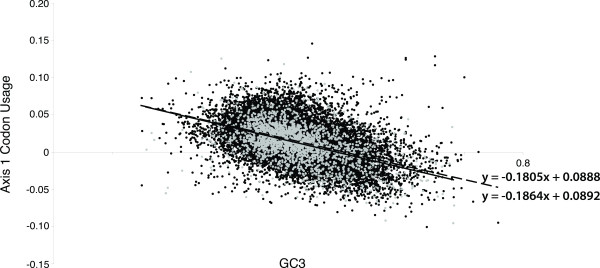
**Synonymous codon usage and codon volatility compared with the composition of GC at the third position.** Codon usage bias, the major contributing axis (Axis 1), is compared to the composition of G and C at the 3rd position of codons. Those genes with significant codon volatilities are overlayed in pale grey. The linear regression for codon usage compared to GC3 composition is shown as the solid line, R^2^ = 0.2566 (equation shown on graph, y = −0.1805x + 0.0888). The linear regression for the codon usage compared to GC3 composition for those genes with significant volatility scores are shown as a dashed line, R^2^ = 0.2281 (equation shown on graph, y = −0.1864x + 0.0892).

Mutational biases have been defined as the systematic asymmetries or nonuniformities in the occurrence of heritable mutations [63]. One such mutational bias that we have focused on in this study is a bias in the frequency at which mutations affect different codons, and as such can affect the ratio of dN to dS (dN/dS). Mutational bias results in an accelerated rate of amino acid replacement in functionally less constrained regions [64]. GC mutation bias deeply influences the folding stability of proteins, making proteins on average less hydrophobic and therefore less stable with respect to unfolding but also less susceptible to misfolding and aggregation [65]. While it has been argued that GC content is correlated with mutational bias in mammalian genes [39], this has not been conclusively shown for *A. thaliana*. To determine if mutational bias could be the driving force for the observed codon usage and codon volatility patterns in this species, we examined the composition of G and C at the third position of *A. thaliana* genes (GC3 composition). GCUA was used to analyse the set of 18,594 genes (only genes of length 100 bp or greater were considered) [47]. By comparing GC3 composition against (i) codon usage values from Axis 1, and (ii), the significant codon volatility scores, a clear correlation is evident between both GC3 composition and codon usage, and also between GC3 composition and codon volatility (Figure 3). No significant difference was found between the R^2^ values for the linear regression line for codon usage (solid line) or codon volatilities (dashed line), which corresponded to values of 0.2566 and 0.2281 respectively. Our results demonstrate that both the patterns of codon usage bias, and similarly the significant codon volatility values, observed for *A. thaliana* are largely an effect of the GC content at the third position of the codon.

## Discussion

Selective pressures operating on amino acid substitutions are typically measured by comparing homologous DNA sequences; (i) from different individuals within a species using polymorphism studies, or (ii), across different species via phylogenetic analysis. In 2004, Plotkin *et al.* proposed a metric to detect selection on the basis of a single genome, dubbed ‘codon volatility’ [12]. While the validity of the codon volatility metric as a surrogate for positive Darwinian selection has been firmly challenged [15-22,24], it is clear that codon volatility values can differ significantly within and between genomes, and investigations continue in order to elucidate the biological basis for this variation. Such studies have focused both on identifying differences in codon volatility trends between the genomes of organisms from diverse taxa, and in attempting to elucidate general trends, whether directly related to selective forces or not. In this study, we have identified the 2,181 most volatile genes in the *A. thaliana* genome and have investigated whether elevated volatility is correlated with dN/dS values indicative of rapid evolutionary rates at these loci, or whether such volatility is better explained by correlation with codon usage bias, differences in gene expression levels, or mutational bias.

Codon volatility measures cannot be used as a proxy for identification of selective pressures in the *A. thaliana* genome as there is no concordance between elevated volatility scores and high dN/dS ratios (Figure 1). Two of the elevated volatility candidate genes had paralogs with depressed volatility. Two genes that have similar functions to their volatile paralogs are described in Table 2. At the nucleotide level At4g15430 and paralog At3g21620 are 84% identical (BLASTN, TAIR), and At4g31590 and paralog At2g24630 are 85% identical, yet these paralogous pairs have codon volatility P-values at opposite ends of the range.

The translational selection model of codon usage bias would predict a correlation between codon usage bias and patterns of gene expression. Indeed, earlier work on codon usage and expression patterns in the *A. thaliana* genome identified a correlation between gene expression levels and codon usage bias (as would be predicted by a translational selection model [35]). However, our analysis conducted with all of the gene models in the *A. thaliana* genome did not find any significant correlation between codon usage bias and gene expression levels (Figure 2B) and does not support a major role for translational efficiency as a driver of codon usage bias in the *A. thaliana* genome. Similarly, genome-wide comparison of codon volatility scores and relative synonymous codon usage values indicated that the significant codon volatility genes are contained within the normal codon usage distribution (Figure 2A).

Earlier studies on codon usage bias and expression levels in *A. thaliana* have suggested that the difference in codon usage between differentially expressed genes can be due to mutation biases and context dependency of codons [38]. Morton and Wright (2007) demonstrated that the variation in codon usage in *Arabidopsis thaliana* is not due to selection, but rather is the result of mutational biases [38]. In rice, codon usage bias in rice has been found to be affected mostly by the genome nucleotide environment, when compared to two other possible factors considered i.e. possible effect of gene expression level and CDS length [66]. However, in contrast to *A. thaliana*, it was observed in rice that genes with higher expression levels exhibit a greater degree of codon usage bias. Such genes are typically GC-rich with a preference for C or G at the synonymous position [66]. The composition of GC in a genome has previously been shown to have an effect on synonymous substitution rate in many different species, including *A. thaliana*[67]. Our analysis indicates that GC3 composition is correlated with codon usage in the *A. thaliana* genome (Figure 3).

It is currently not known whether mutational bias has played a significant role in the variation in codon usage between genomes and genes in plants. Contrasting patterns of codon usage have been observed between *A. thaliana* and rice, where rice genes exhibit a wide, multimodal distribution, in comparison to the much narrower, unimodal distribution of codon usage seen for *Arabidopsis* genes [44]. Despite this contrasting pattern, both plant species demonstrate a strong correlation between the nucleotide composition at the third positions (GC3) and codon usage. The increase in the GC content of the subset of the rice genes since the evolutionary divergence of monocot and dicot plants has been suggested as a possible explanation for the multimodal distribution of codon usage in rice genome. Wang and Hickey (2007) suggest that the variation in codon usage among rice genes is due to the mutational bias at the DNA level rather than natural selection acting at the level of mRNA translation [44]. However, Wang & Hickey (2007) also suggest that an absence of strong mutational bias in the *A. thaliana* genome facilitates the detection of translational selection [44] Our results indicate that mutational bias is strongly present in *A. thaliana* to a greater extent than any selection for translational efficiency. In this study, we have determined that GC3 composition, and therefore mutational bias, are the major contributors to the codon usage bias and the codon volatility patterns observed for in the *A. thaliana* genome.

## Conclusion

We set out to determine whether those genes in the *A. thaliana* genome that exhibit the highest codon volatility also had high dN/dS values (indicative of positive selection). We found no correlation between the codon volatility measurements and dN/dS ratios across the *A. thaliana* genome. To determine what possible phenomenon codon volatility was measuring - we compared codon volatility with codon usage biases and GC3 composition across the genome. We found that neither codon volatility nor codon usage is correlated with gene expression values but rather that they both are directly correlated with the composition of GC at the third position of codons. Our analyses clearly indicate that codon volatility does not measure selective pressures in *Arabidopsis thaliana*. Significant codon volatility values for specific genes tend to correspond to those genes with less biased codon usage. Both significant codon volatility *P*-values and codon usage are correlated with GC3 in *A. thaliana* suggesting that mutational bias rather than selection for translational efficiency are driving the evolution of this plant genome.

## Abbreviations

dN: Nonsynonymous substitutions per nonsynonymous site; dS: Synonymous substitutions per synonymous site; dN/dS: Ratio of the number of non-synonymous substitutions per non-synonymous site to the number of synonymous substitutions per synonymous site; GC: Guanine cytosine; GC3: Composition of guanine and cytosine at the third codon position; At: *Arabidopsis thaliana*; Al: *Arabidopsis lyrata*; TAIR: The arabidopsis information resource; P: probability; DNA: Deoxyribonucleic acid; CDS: Coding DNA sequence; K: Kappa; SCR: Self-incompatibility locus; MAM: Methylthioalkylmalate locus; RSCU: Relative synonymous codon usage; TIGR: The Institute for Genomic Research (The J. Craig Venter Institute); nt: nucleotide; PCR: Polymerase chain reaction.

## Competing interests

The author(s) declare that they have no competing interests.

## Authors’ contributions

MJOC carried out the codon usage analysis, selection analysis, codon volatility analysis, all correlational analyses and participated in drafting the manuscript. AMD performed the sequencing, paralogon analysis, GO analyses, was engaged in selection analysis of *A. lyrata* genes and participated in drafting the manuscript. TEJ carried out the expression analyses and participated in drafting the manuscript. CK and RT participated in drafting of the manuscript. MTAD identified the genes in *A. lyrata* and performed the selection analysis for *A. lyrata*. CS was responsible for project design, research and data management, project management, and in drafting and finalising the manuscript. All authors read and approved the final manuscript.

## Supplementary Material

Additional file 1 **Genes with significantly elevated codon volatilities in*Arabidopsis thaliana.*** Click here for file
